# Effects of Storage on Quality Traits of Sausages Made with Chicken Breast Meat Affected by Wooden Breast

**DOI:** 10.3390/ani11020513

**Published:** 2021-02-16

**Authors:** Rodrigo Fortunato de Oliveira, Maísa Santos Fávero, Juliana Lolli Malagoli de Mello, Fábio Borba Ferrari, Erika Nayara Freire Cavalcanti, Rodrigo Alves de Souza, Mateus Roberto Pereira, Aline Giampietro-Ganeco, Erick Alonso Villegas-Cayllahua, Heloisa de Almeida Fidelis, Pedro Alves de Souza, Hirasilva Borba

**Affiliations:** 1 Department of Technology, Universidade Estadual Paulista—UNESP, N/n, Professor Donato Castellane Access Road, Rural Zone, Jaboticabal 14884-900, Sao Paulo, Brazil; maisa.sfavero@gmail.com (M.S.F.); julianalolli@zootecnista.com.br (J.L.M.d.M.); fbf_zoo@hotmail.com (F.B.F.); mateusscj2012@hotmail.com (M.R.P.); erikanayarac@gmail.com (E.N.F.C.); eav.cayllahua@unesp.br (E.A.V.-C.); heloisa.a.fidelis@gmail.com (H.d.A.F.); p.souza@unesp.br (P.A.d.S.); 2 Faculty of Animal Science and Food Engineering, 225, University of São Paulo—USP, Duque de Caxias Norte Avenue, Pirassununga 13635-900, Sao Paulo, Brazil; rodrigo.zootecnista@gmail.com (R.A.d.S.); giampietroganeco@gmail.com (A.G.-G.)

**Keywords:** chicken breast meat, meat quality, muscle disease, cooling storage, wooden breast

## Abstract

**Simple Summary:**

Studies of the meat quality of modern birds and their respective myopathies are important to clarify the influence of these on the meat quality of the animals. The constant genetic evolution that birds have undergone is the most plausible cause for the onset of Wooden Breast Myopathy. The processing of byproducts, such as sausages, represents an alternative to avoid the losses that this myopathy generates to the poultry industry due to discards of chickens affected by different degrees of wooden breast myopathy.

**Abstract:**

The aim of this study was to examine the effects of storage on the quality of sausages made with breast from chickens affected by wooden breast myopathy (WBM). Breast samples from male broilers slaughtered at 48 days old were used. Normal (absence of myopathy), moderate degree (hardness only in one region of the breast) and severe degree samples (hardness over the entire length of the breast) were processed into sausages and evaluated prior to storage and after being vacuum-packed and stored for 7, 14, 21 and 28 days at 4 °C. There was a decrease (*p* < 0.001) in pH and an increase (*p* < 0.001) in cooking weight loss in samples of sausages, regardless of the myopathy, after 28 days of storage. Sausages produced with chicken breast samples affected by wooden breast myopathy presented higher (*p* < 0.0001) moisture concentration (72% for the severe degree) and higher (*p* = 0.0224) protein concentration (17.27% and 17.36%, respectively, for the moderate and severe degrees) than sausages made of normal samples (70.72% and 14.32%, respectively). The results indicate that sausages produced with meat from birds moderately and severely affected by the myopathy show higher oxidative stability. Fresh sausages produced with breast meat from birds affected by wooden breast syndrome may be stored (4 °C) for up to 28 days without exhibiting the characteristic rancid taste and smell. In sensory analysis, no differences were observed between the formulations, which suggests that the consumers approved the samples regardless of the disease severity in the meat used for the making of the sausages. The current results show that chicken meat affected by wooden breast myopathy can be used for producing fresh sausages in the industry.

## 1. Introduction

The world’s chicken meat production has grown markedly in the last decades, with a 3% increase estimated for 2020 in relation to the 95.5 million tons produced in 2019 [1]. Today’s successful production and exports of chicken meat are a result of measures adopted by the poultry industry, with high growth rates, higher feed efficiency ratios and elevated meat production [2] that meet the market demands. However, problems that compromise the functionality and quality of chicken meat have accompanied genetic selections [3], and one of the serious problems affecting chicken meat quality is the wooden breast myopathy (WBM) [4]. This disease, whose etiology is unknown, leads to changes in appearance, decrease in technological and nutritional quality and, consequently, changes in consumer acceptance [5,6,7,8,9]. In this sense, pectoralis major muscles affected by WBM are heavier and thicker than unaffected muscles and exhibit partially or entirely hardened muscle regions and surfaces covered by a turgid, viscous fluid. In rare cases, bleeding may also be present [8,10]. The myopathy can only be detected manually during inspection [8]. Wooden breast has been reported in many parts of the world, such as Europe and the USA [6,7,8,11], and its incidence has increased as a result of the increasing growth and meat-yield rates of modern broiler chicken hybrids required by the industry. Moreover, the breast meat affected by this disorder shows higher cooking weight loss [12]. However, to date, it is not known whether this decline in meat quality influences other functional properties that may interfere with the production of sausage products.

The wooden breast abnormality is characterized by pale breasts and breasts with hardened areas, exudation and bleeding, and sometimes, wooden breast may be associated with other myopathies, such as white striping along the muscle surface [8,13]. WBM may be found at different severity degrees [4} and it provides a hardened meat with an unappealing color to the consumer. Therefore, the meat affected by this condition no longer meets the sensory standards for direct sale, and it is used for animal feed or discarded [14].

However, this meat may be used for the manufacturing of processed meat products [6,15]. Fresh sausages emerge as an option for the technological utilization of chicken breast affected by WBM, given its commercial value. In this context, Brazilian law provides for the use of breasts affected by WBM in the production of industrialized meat products [16]. Nevertheless, there is a lack of data on the storage of chicken sausages prepared with meat affected by the disorder. In this context, studies investigating the effects of storage time and conditions of meat products are essential for the processing of these products, since problems related to storage stability are common [17].

On this basis, the purpose of the present study was to evaluate the possible effects of storage on the quality of fresh sausages made with breast meat from chickens affected by different degrees of wooden breast myopathy.

## 2. Materials and Methods 

This study was conducted in the Laboratory of Analysis of Animal Origin Foods of the Faculty of Agrarian and Veterinary Sciences of Paulista State University (UNESP), Campus Jaboticabal, São Paulo, Brazil (21°08′ S, 48°11′ W, 583 m altitude).

### 2.1. Sample Collection and Experimental Procedure

The chickens were reared in a traditional intensive system and slaughtered at 48 days old. Muscle samples of pectoralis major affected by WBM were collected during one sampling session from male Ross chickens (AP 95) in a commercial slaughterhouse (SP, Brazil) inspected by the Federal Inspection Service. The samples were classified according to Tijare et al. and Sihvo et al. [2,8] and according to the severity degree of the myopathy (moderate—hardness found only in the cranial region or caudal region of the breast fillet; severe—hardness found over the entire length of the breast fillet) and control samples (samples without myopathies) (10 ± 1.3 kg per group). After deboning and characterization (one hour postmortem) of the myopathy’s severity degree, the samples were separated within each anomaly category and transported to the university laboratory under cooling conditions (±4 °C).

Four hours after slaughter (establishment of rigor mortis), the samples collected from each evaluated group after connective tissue discard were cut into cubes and ground in an Eberle manual meat grinder with a 5 mm disc. In the sausage formulation (Table 1), chicken skin was added to reach 10% of fat percentage. After grinding, the ingredients were added and thoroughly mixed in a mixer. The chicken mix obtained was encased in natural pig intestines obtained from UNESP using a manual 36 mm sausage funnel.

After processing, the samples were separated (six sausages in each bag) and vacuum-packed (Selovac 200-B, São Paulo, Brazil) in plastic bags (18 µ), weighed and stored in a BOD (Biological Oxygen Demand) incubator (Eletrolab EL101/3 250W, Eletrolab, São Paulo, Brazil) at 4 ± 0.5 °C. The physicochemical analyses described next were performed on non-stored samples (on the collection day; *n* = 20 for each severity degree and control group (total *n* = 60)) and after 7, 14, 21 and 28 days of storage (*n* = 20 for each degree of severity and control group (total *n* = 60), for each period).

### 2.2. Methods

The chemical composition was determined after physical analyses were performed. Raw samples were freeze-dried (SuperModulyo220, Thermo Fisher Scientific Inc., Waltham, MA, USA) and ground to subsequently determine the protein and mineral matter concentrations as recommended by [18] methods 977.14 and 920.153, respectively. The moisture percentage was determined as the difference between the weights of the samples before and after freeze-drying (16, method 950.46). Fat was determined by following the method proposed by [19]. 

The color was determined using the Minolta Chrome Meter CR-400 (Konica Minolta Sensing, Inc., Osaka, Japan) colorimeter (settings: diffused lighting/0 viewing angle, illuminant D65, specular component included) calibrated to a white standard, using the CIELAB system (L*, a* and b*). Parameters, such as lightness (L*), redness (a*) and yellowness (b*), were evaluated at three different positions on the surface of raw cut in half. The pH was measured in triplicate with a digital pH meter (Testo 205, Testo Inc., Sparta, NJ, USA) equipped with a penetration electrode, which was inserted directly into each sample.

Raw chicken sausage sub-samples were inserted into the container (with a capacity of 0.015 l) of Aqualab (Decal Devices Inc., Brooklyn, NY, USA) water activity analyzer (Aw), which uses the dew point principle [18]. 

Weight loss during storage was defined as the difference between the final and initial weights of each sample before and after the storage period. Results for this variable were expressed in percentage terms. 

Before cooking, the samples were weighed on an analytical scale. Subsequently, they were cooked on a preheated (10 min) grill (George Foreman GBZ80, Orlando, FL, USA) until the inner temperature of the samples reached 85 °C (temperature controlled by stainless steel thermocouples that were inserted individually into each sample; FE-MUX, Flyever Indústria e Comércio de Equipamentos Eletrônicos Ltd., São Carlos-SP, Brazil), according to the method proposed by [20]. After cooling at room temperature, the samples were weighed again to determine cooking weight losses (CWL). The results of this variable were obtained as the difference between the final and initial weights and expressed in percentage. From each cooked sample, three cylindrical subsamples, with known area (6.25 cm diameter and 1.5-cm thick) were cut for the performance of hardness analysis on a texturometer (TAXT2i, Stable Micro Systems, LTD., Godalming, UK) fitted with a compression plate (P/75, 75-mm aluminum platen) using the following settings: pretest speed 2.0 mm s^−1^; post-test speed 5.0 mm s^−1^; strain 50%; time 5.0 s; trigger type, auto; and trigger force 5 g. The results were expressed in Newtons (N).

The lipid oxidation was determined in all raw samples by the thiobarbituric acid reactive substances (TBARs) test, according to the methodology described by [21], which uses trichloroacetic acid for the extraction of 0.005 kg of ground sample. After reaction under heating with thiobarbituric acid, a reading at the 538 nm wavelength was performed, and the result is expressed in malonaldehyde mg (MDA)/kg. An acceptance test with hedonic scale was used for the sensory evaluation, using a scale of 9 scores (9 = extremely like, 5 = neither like nor dislike, and 1 = extremely dislike) commonly used for meat evaluation [22]. The sausages were rated for sensory attributes by 120 untrained participants (80 women and 40 men, aged between 18 and 45 years old), who were recruited among students and staff members of UNESP, Jaboticabal Campus. Briefly, 1.5 cm thick slices were cut, starting from the middle of the cooked sausage. One slice of cooked sausage from each severity degree of the myopathy was coded with three-digit numbers, and their order was randomized for each evaluator. Water and unsalted crackers were provided for cleansing the palate between evaluations. Participants were given an evaluation form with the nine-score hedonic scale to evaluate aroma, flavor, texture, appearance and overall acceptance. The evaluations were carried out in individual booths. A consumer test was conducted after verifying the compliance of the microbiological parameters established by the Brazilian legislation for total and thermotolerant coliforms at 45 °C/kg, *Salmonella* spp./0.025 kg, coagulase-positive Staphylococci/g, mesophilic bacteria/kg and psychotrophic bacteria/kg [23]. The study followed the ethical requirements of the sensory laboratory approved by the Paulista State University research ethics committee on 23rd September 2020 (Ethic code: 4.294.459/CAAE 33849720.1.0000.9029), and informed consent was signed by the panelists. 

### 2.3. Statistical Analysis

Statistical analyses were performed using the SAS statistical software (9.4). All data were submitted to normality analysis using the Shapiro–Wilk test at a 5% probability level. The dependent variables that did not present normal distribution were normalized through PROC RANK of the SAS statistical software (9.4). The data obtained from the physicochemical analyses were analyzed through a completely randomized experimental design (CRD) in a 3 × 5 factorial scheme (three myopathy severity degrees and five storage periods) with 20 replications. The data obtained in the chemical composition and consumer test were analyzed through a completely randomized experimental design (CRD) with 20 and 120 replications, respectively. All data were tested by analysis of variance (ANOVA) and compared by Tukey test at a significance level of 5%.

## 3. Results

### 3.1. Chemical Composition

There was an interaction effect (*p* = 0.0053) between disease severity and storage time for the protein content of the samples (Table 2). Sausages freshly produced with chicken meat affected by the myopathy had less (*p* = 0.0224) protein content than sausages produced with normal chicken meat. After 7 and 21 days of storage, sausages produced with chicken meat affected by the myopathy had greater protein content than sausages produced with normal chicken meat. There was an effect (*p* < 0.0001) of storage on the protein content of sausages produced with chicken meat affected by the myopathy, which reduced the protein content up to 28 days of storage. While both disease severity (*p* < 0.0001) and storage time (*p* = 0.0003) influenced moisture percentage, neither the fat nor the ash content in the fresh chicken sausage changed (*p* > 0.05).

Samples produced with meat from birds severely affected by the myopathy showed greater moisture than those made with meat from normal birds and from birds moderately affected by the condition. There was a decrease in sample moisture after 21 days of storage, regardless of the disease condition.

There was no significant effect (*p* > 0.05) of disease severity or storage on the fat concentration in the sausages.

### 3.2. Weight Loss during Storage, Water Activity and Lipid Oxidation

There was a significant interaction (*p* < 0.05) between disease severity and storage period for weight loss during storage, Aw and lipid oxidation (Table 3). Samples produced with meat from birds affected by the severe degree of the myopathy showed higher (*p* < 0.0001) weight losses (Table 4) after 14 and 21 days of storage due to their lower water holding capacity. After 21 days of storage, weight loss in the fresh sausages increased from 3.21% (normal samples) to 4.21% (severely affected samples).

Sausages produced with chicken meat affected by the myopathy had higher (*p* < 0.0001) Aw than sausages produced with normal chicken meat after seven days of storage. There was an effect (*p* < 0.0001) of storage on the Aw of sausages produced with breast meat, regardless of the myopathy, which reduced its value up to 28 days of storage. Compared to the samples produced with meat from normal chickens, those made with meat from birds affected by the severe degree of WBM showed lower variations in lipid oxidation values after 28 days of cooling (0.42 to 0.62 mg MDA/kg). In the normal-meat samples, lipid oxidation rose from 0.38 to 1.29 mg MDA/kg in the same period.

### 3.3. Sausage Color

There was a significant interaction effect (*p* < 0.05) between disease severity and storage time for lightness (L*), redness (a*) and yellowness (b*) values in the samples of fresh chicken sausage (Table 4). 

Samples affected by the severe degree of WBM exhibited an increase (*p* = 0.0008) in the L* value compared to normal samples (except for 7 and 28 days of storage). During storage, both the samples made with breast meat from normal birds and meat from chickens affected by the myopathy showed an increase (*p* < 0.0001) in the L* value, with maximum lightness achieved at 21 days. Samples affected by the severe degree of wooden breast myopathy showed an increase (*p* = 0.0122) in the a* value compared to normal samples (except for 7 and 28 days of storage). During storage, both sausage samples made with normal breasts and breasts affected by moderate and severe myopathy degree showed an increase (*p* < 0.0001) in a* value up to 21 days of storage, and afterwards, all samples showed a decrease after 28 days of storage. Regarding the yellowness, sausage samples not stored and stored for 14 and 21 days and produced with breast affected by a severe degree of myopathy presented higher b* value (9.63, 10.48 and 13.25, respectively) than samples produced with normal breast (8.49, 8.72 and 11.53, respectively) or those produced with the breast affected by the moderate degree (7.87, 7.61 and 11.46, respectively).

### 3.4. pH, Cooking Weight Loss and Tenderness

There was a significant interaction (*p* < 0.0001) between disease severity and storage time for pH and CWL in the samples of fresh chicken sausage (Table 5). Sausages produced with chicken meat affected by the myopathy had higher (*p* < 0.0001) pH than sausages produced with normal chicken meat. There was an effect (*p* < 0.0001) of storage on the pH of sausages produced with breast meat, regardless of the myopathy, which decreased the pH value up to 28 days of storage. 

Samples made with meat from chickens affected by WBM showed higher CWL (*p* < 0.0001) than those made with normal meat. 

Regarding the hardness, no significant interaction was detected (*p* > 0.05) between disease severity and storage period. Normal samples and samples affected by the moderate degree of the disease were tougher (*p* = 0.0021) when compared to those made with meat from birds affected by the severe degree of the disease. 

### 3.5. Sensory Analysis

The parameters evaluated in the sensory analysis (Table 6) performed by untrained tasters did not significantly differ (*p* > 0.05).

## 4. Discussion

The higher moisture observed in the sausages made with meat from birds severely affected by WBM was due to the moisture percentage of the raw material [9,24,25], as it shows more moisture than healthy chicken breasts without processing. Reference [8] explained that the increased moisture in breasts affected by WBM may be a result of the presence of breast edema as a consequence of inflammatory processes. In general, all products lost moisture during storage due to exudate production, which may explain the weight loss (Table 3).

Variations in protein percentage of sausages may be related to the lack of uniformity, although WBM was expected to present a decreasing protein content due to the notable changes in muscle structure that are characteristic of the pectoralis major muscles affected by WBM [9,12]. Studies have shown that the occurrence of wooden breast negatively affects some aspects of meat, including its histology, nutritional and chemical composition and technological parameters [13]. The myodegenerative processes in muscles affected by wooden breast cause changes in the water-holding capacity and induce low levels of protein [9], which directly affects the processing of chicken meat [2,6,8,9]. Regardless of disease severity, the Aw value in the samples was not preserved throughout storage for 28 days under cooling. Low-temperature storage is the indicated method to preserve the quality of products considered perishable, since these foods have higher Aw values, which decrease when subjected to the low-temperature storage process. In chicken sausages, regardless of the occurrence or absence of myopathy, lower values of water activity were observed in products that underwent cooling in different periods of analysis, reducing the chances of suffering microbial action. These results corroborate those presented by [14], who froze hamburgers produced with chicken breasts affected by wooden breast myopathy and observed a decrease in Aw after 120 days of storage. 

Based on the results shown in Table 3, we observed that the lipid oxidation was low, which was likely due to the addition of sodium nitrite in a fresh product. Reference [26] demonstrated that under those experimental conditions, a TBARS value around 2 mg MDA/kg could be considered the threshold for the acceptability of oxidised beef. The high lipid oxidation found in products made with chicken meat is due to the greater susceptibility to the characteristic oxidation of polyunsaturated fatty acids present in this type of raw material [27].

Considering the maximum value of 2 mg MDA/kg per sample for rancidity to be perceived, the storage of fresh sausages prepared either with or without meat from birds affected by WBM under cooling for up 28 days prevent the occurrence of rancidity. Reference [9] found higher TBARS values in breasts affected by the disorder and attributed this result to the amount of fat present in the studied samples. No such result was observed in the affected samples in the present study, where the fat content did not differ between the samples. The TBARS results of this study may also be explained by the water activity of the products (Table 3), being that a decrease in water activity promotes a higher lipid oxidation in them. 

In processed products whose formulation includes the addition of curing salt (sodium nitrite), as is the case of sausage, a more uniform color is commonly observed, since the curing process in meat products reduces the a* value, increases the b* value and may or may not alter L [28]. As a result, the product color is changed to hazel or brown [28], which was demonstrated in this study by the increased b* value found in all samples.

The increase in luminosity according to the storage time may be a result of a lower volume of muscle proteins (Table 2) and consequently greater spacing between the filaments, which generates greater light scatter as a result of muscle fiber degeneration [13], mainly in samples produced with chicken breasts affected by wooden breast myopathy. This finding is in accordance with the results published by Ref. [29] in a study that revealed that L* values tend to increase with time. 

High a* values may be associated with storage and packaging methods. In the present study, the vacuum-packaging and cooling storage methods used prevented the contact of meat with oxygen and the occurrence of reactions that would have affected the color. Similar results were presented by [30], who worked with vacuum packaging in storage and obtained an increase in a* values.

The prolonged frozen storage can promote oxidative processes, which can influence the product color (mostly lightness and yellowness) [14]. As the sausage cooling storage in this experiment was short (28 days), the oxidative processes that promote color reduction (mostly lightness and yellowness) may not have had the expected effect. These high pH values in products (sausages and hamburgers) produced with chicken meat affected by wooden breast myopathy indicate that there was an influence of the pH value of raw meat, since the low glycolysis presented after slaughter in breast affected by this myopathy results in a higher pH [14]. In particular, the higher final pH found in fillets with wooden breast myopathy is probably due to the decreased glycolytic potential, energy status and changes in metabolic pathways in the muscles of birds presenting such myopathy [24,31].

Cooking weight loss is considered to be one of the most important functional properties of meat products [32]. The samples made with meat from chicken severely affected by WBM and stored for 28 days showed lower CWL than the samples produced with normal meat and with meat from birds moderately affected by the myopathy. Cooking weight losses increased until 28 days of storage in all studied samples. The increasing CWL in the samples produced with meat from birds affected by WBM may be explained by defects in myofibrillar or sarcoplasmic proteins or histological changes that occur in the meat [6,9,33]. Reference [15] observed that, in comminuted products made with breasts affected by WBM, CWL was low and close to those of products formulated with breast meat from normal birds, which was a consequence of the formation of a three-dimensional network between proteins and fat in ground meat. Kozačins et al. [17] evaluated hamburgers produced with chicken meat affected by wooden breast myopathy and found no influence on cooking weight loss; similar results were found by [14], who observed no influence on CWL in hamburgers produced with chicken meat affected by WBM after 120 days of storage. The authors also suggest that the grinding of fillets affected by wooden breast myopathy may reduce the negative influence of the myopathy condition on the cooking weight loss of breast meat. Reports show that, after storage, cooking weight loss of meat affected by wooden breast myopathy is higher than normal breast [2] due to the lower water holding capacity. These results indicate that the wooden breast myopathy has a negative effect, increasing the losses (CWL) up to 7 days of storage for the moderate degree and 28 days for the severe degree. 

The storage process resulted in decreased tenderness in the samples. The decreased tenderness observed in the severely affected samples might have been caused by fiber degeneration and reduced salt-soluble myofibrillar protein content [6] as well as by the amount of fat content, which did not significantly differ among groups. The results of sensory analysis indicate the potential use of chicken breast affected by WBM in the making of fresh sausages, since the tasters did not detect differences between the sensory characteristics of products made with normal chicken breast and chicken breast affected by moderate and severe degrees of the disease. The acceptance indices for appearance, aroma, taste, texture and overall acceptance were higher than 6.0, which indicates that the samples were sensorily accepted. Specifically regarding the taste, all samples had a good overall acceptance and received an average score of 7.58 (really liked).

## 5. Conclusions

Although the samples of fresh sausage produced with breast meat from chickens affected by wooden breast myopathy exhibited acceptable chemical characteristics, the raw material used in the product elaboration caused a slight decrease in physical quality in terms of color, pH and cooking loss. Cooling storage for 28 days had a positive effect on sausage quality produced with chicken meat affected by the disease, mainly indicated by chemical composition, weight loss during storage, lipid oxidation, color (L*, a*, b*) and cooking weight loss, quality parameters that did not vary between samples or showed little variation after 28 days of storage. Fresh sausages produced with breast meat from birds affected by WBM can be stored (4 °C) for up to 28 days without exhibiting the characteristic taste and smell of rancidity. Chicken breast affected by the wooden breast myopathy showed great potential to be used in the making of fresh sausage since the sensory parameters of the product were not influenced by the addition of affected meat. The current results demonstrate that chicken meat affected by wooden breast myopathy can be used in the industry in the making of fresh sausages. 

## Figures and Tables

**Table 1:** Formulation used for sausages production.

Ingredients	(%)
Breast filet	85.0
Chicken skin	10.0
Water	2.0
Salt	1.5
Garlic paste	0.5
Ground white pepper	0.15
Citric acid (antioxidant)	0.735
Sugar	0.095
Sodium nitrite	0.02
Total	100

**Table 2:** Mean values of moisture, total lipids and protein concentrations in fresh sausages produced with breast meat from Ross (AP95) broiler chickens without myopathies and affected by wooden breast myopathy after different storage periods.

	Moisture (%)	Total Lipids (%)		Ashes (%)	
Severity degree (SD)					
Normal	70.72 ± 0.11 ^B^	4.84 ± 1.17		2.36 ± 0.11	
Moderate	70.96 ± 0.10 ^B^	5.33 ± 1.97		2.44 ± 0.10	
Severe	72.00 ± 0.09 ^A^	5.57 ± 1.13		2.54 ± 0.09	
Storage time (ST)					
Non-stored	71.55 ± 0.15 ^A^	4.79 ± 1.12		2.44 ± 0.09	
7 days	71.45 ± 0.12 ^A^	5.01 ± 1.18		2.43 ± 0.10	
14 days	71.34 ± 0.12 ^A^	5.26 ± 1.18		2.42 ± 0.10	
21 days	70.87 ± 0.12 ^B^	5.08 ± 1.16		2.42 ± 0.12	
28 days	70.93 ± 0.12 ^B^	6.10 ± 1.17		2.51 ± 0.09	
*p*-value					
(SD)	<0.0001	0.117		0.1702	
(ST)	0.0003	0.0858		0.1135	
(SD × ST)	0.2419	0.4941		0.0889	
Protein (%)					
ST (*n* = 20) ^1^	SD (*n* = 20) ^2^			*p*-Value	
	Normal	Moderate	Severe		
Non-stored	19.72 ± 0.61 ^Aa^	18.76 ± 0.61 ^Aab^	17.90 ± 0.61 ^Ab^		
7 days	14.32 ± 0.64 ^Bb^	17.27 ± 0.61 ^ABa^	17.36 ± 0.58 ^ABa^	(SD)	0.0224
14 days	15.47 ± 0.61 ^Ba^	16.19 ± 0.61 ^BCa^	15.94 ± 0.61 ^BCa^	(ST)	<0.0001
21 days	14.37 ± 0.61 ^Bb^	17.05 ± 0.61 ^BCa^	16.37 ± 0.58 ^ABCa^	(SD × ST)	0.0053
28 days	15.48 ± 0.64 ^Ba^	15.47 ± 0.69 ^Ca^	15.45 ± 0.58 ^Ca^		

^1 A,B^ Means followed by different capital letters in the same column differ from each other by the Tukey test (*p* < 0.05). ^2 a–c^ Means followed by different lowercase letters on the same line differ from each other by the Tukey test (*p* < 0.05). ST—storage time/20 samples/group, SD—severity degree/20 samples/group.

**Table 3:** Mean values of weight loss during storage, water activity (Aw) and lipid oxidation (TBARS) in fresh sausages produced with breast meat from Ross (AP95) broiler chickens without myopathies and affected by wooden breast myopathy after different storage periods.

Weight Loss During Storage (%)					
ST (*n* = 20) ^1^	SD (*n* = 20) ^2^			*p*-Value	
	Normal	Moderate	Severe		
7 days	3.40 ± 0.15 ^Ba^	3.15 ± 0.15 ^Ca^	3.43 ± 0.15 ^Ba^	(SD)	<0.0001
14 days	3.21 ± 0.15 ^Bb^	3.55 ± 0.15 ^Bb^	4.04 ± 0.15 ^Aa^	(ST)	<0.0001
21 days	3.21 ± 0.15 ^Bb^	4.02 ± 0.15 ^Aa^	4.21 ± 0.15 ^Aa^	(SD × ST)	0.0008
28 days	4.06 ± 0.15 ^Aa^	4.25 ± 0.15 ^Aa^	4.04 ± 0.15 ^Aa^		
Aw					
ST (*n* = 20) ^1^	Normal	Moderate	Severe	*p*-Value	
Non-stored	0.972 ± 0.002 ^Aab^	0.968 ± 0.002 ^Ab^	0.973 ± 0.002 ^Aa^		
7 days	0.925 ± 0.002 ^Bc^	0.931 ± 0.002 ^Bb^	0.946 ± 0.003 ^Ba^	(SD)	<0.0001
14 days	0.890 ± 0.002 ^Ec^	0.916 ± 0.002 ^Ca^	0.901 ± 0.002 ^Eb^	(ST)	<0.0001
21 days	0.902 ± 0.002 ^Db^	0.900 ± 0.002 ^Eb^	0.908 ± 0.002 ^Da^	(SD × ST)	<0.0001
28 days	0.911 ± 0.002 ^Cb^	0.909 ± 0.002 ^Db^	0.919 ± 0.002 ^Ca^		
TBARS (mg MDA/kg)					
ST (*n* = 20) ^1^	Normal	Moderate	Severe	*p*-Value	
Non-stored	0.38 ± 0.04 ^Da^	0.37 ± 0.04 ^Ca^	0.42 ± 0.04 ^Da^		
7 days	0.87 ± 0.05 ^Bb^	0.94 ± 0.05 ^Ab^	1.11 ± 0.04 ^Aa^	(SD)	<0.0001
14 days	1.39 ± 0.05 ^Aa^	0.89 ± 0.05 ^Ab^	0.70 ± 0.04 ^Cc^	(ST)	<0.0001
21 days	0.66 ± 0.04 ^Cb^	0.67 ± 0.04 ^Bb^	0.84 ± 0.04 ^Ba^	(SD × ST)	<0.0001
28 days	1.29 ± 0.05 ^Aa^	0.59 ± 0.04 ^Bb^	0.62 ± 0.04 ^Cb^		

^1 A–E^ Means followed by different capital letters in the same column differ from each other by the Tukey test (*p* < 0.05). ^2 a–c^ Means followed by different lowercase letters on the same line differ from each other by the Tukey test (*p* < 0.05). ST—storage time/20 samples/group, SD—severity degree/20 samples/group.

**Table 4:** Lightness (L*), redness (a*) and yellowness (b*) mean values in fresh sausages produced with breast meat from Ross (AP95) without myopathies and affected by wooden breast myopathy after different storage periods.

L*					
ST (*n* = 20) ^1^	SD (*n* = 20) ^2^			*p*-Value	
	Normal	Moderate	Severe		
Non-stored	54.27 ± 0.57 ^Eb^	55.93 ± 0.57 ^Db^	58.72 ± 0.56 ^Ca^		
7 days	60.89 ± 0.59 ^Ca^	56.84 ± 0.59 ^Db^	59.26 ± 0.61 ^Ca^	(SD)	0.0008
14 days	59.22 ± 0.59 ^Db^	60.44 ± 0.59 ^Cb^	62.12 ± 0.59 ^Ba^	(ST)	<0.0001
21 days	67.47 ± 0.59 ^Ab^	68.56 ± 0.59 ^Aab^	69.29 ± 0.59 ^Aa^	(SD × ST)	<0.0001
28 days	62.07 ± 0.59 ^BCa^	62.35 ± 0.62 ^Ba^	60.85 ± 0.59 ^BCa^		
a*					
ST (*n* = 20) ^1^	Normal	Moderate	Severe	*p*-Value	
Non-stored	1.54 ± 0.09 ^Cb^	1.48 ± 0.09 ^Eb^	1.93 ± 0.09 ^CDa^		
7 days	2.17 ± 0.09 ^Ba^	1.84 ± 0.09 ^Db^	1.71 ± 0.10 ^Db^	(SD)	0.0122
14 days	2.25 ± 0.09 ^Bb^	2.68 ± 0.10 ^Ba^	2.58 ± 0.09 ^Ba^	(ST)	<0.0001
21 days	2.89 ± 0.10 ^Ab^	2.94 ± 0.09 ^Ab^	3.46 ± 0.10 ^Aa^	(SD × ST)	<0.0001
28 days	2.03 ± 0.09 ^Ba^	2.28 ± 0.11 ^Ca^	2.07 ± 0.09 ^Ca^		
b*					
ST (*n* = 20) ^1^	Normal	Moderate	Severe	*p*-Value	
Non-stored	8.49 ± 0.21 ^Cb^	7.87 ± 0.22 ^CDb^	9.63 ± 0.21 ^Ca^		
7 days	10.00 ± 0.23 ^Ba^	10.00 ± 0.23 ^Ba^	9.66 ± 0.23 ^Ca^	(SD)	0.0319
14 days	8.72 ± 0.22 ^Cb^	7.61 ± 0.22 ^Db^	10.48 ± 0.22 ^Ba^	(ST)	<0.0001
21 days	11.53 ± 0.25 ^Ab^	11.46 ± 0.22 ^Ab^	13.25 ± 0.24 ^Aa^	(SD × ST)	0.0159
28 days	9.79 ± 0.22 ^Ba^	8.29 ± 0.24 ^Cb^	9.35 ± 0.22 ^Ca^		

^1/A–E^ Means followed by different capital letters in the same column differ from each other by the Tukey test (*p* < 0.05). ^2/a–b^ Means followed by different lowercase letters on the same line differ from each other by the Tukey test (*p* < 0.05). ST—storage time/20 samples/group, SD—severity degree/20 samples/group.

**Table 5:** Mean values of pH and cooking loss in fresh sausages produced with breast meat from Ross (AP95) broiler chickens without myopathies and affected by wooden breast myopathy after different storage periods.

pH					
ST (*n* = 20) ^1^	SD (*n* = 20) ^2^			*p*-Value	
	Normal	Moderate	Severe		
Non-stored	5.855 ± 0.004 ^Bc^	5.927 ± 0.004 ^Ab^	5.952 ± 0.004 ^Aa^		
7 days	5.775 ± 0.004 ^Cc^	5.832 ± 0.004 ^Bb^	5.919 ± 0.004 ^Ba^	(SD)	<0.0001
14 days	5.894 ± 0.004 ^Ab^	5.918 ± 0.004 ^Aa^	5.858 ± 0.004 ^Cc^	(ST)	<0.0001
21 days	5.616 ± 0.004 ^Eb^	5.689 ± 0.005 ^Ca^	5.611 ± 0.004 ^Db^	(SD × ST)	<0.0001
28 days	5.564 ± 0.004 ^Dc^	5.590 ± 0.004 ^Db^	5.608 ± 0.004 ^CDa^		
Cooking weight loss (%)					
ST (*n* = 20) ^1^	Normal	Moderate	Severe	*p*-Value	
Non-stored	22.41 ± 0.90 ^Cb^	28.12 ± 0.92 ^Ba^	27.45 ± 0.90 ^Da^		
7 days	24.80 ± 0.90 ^BCb^	24.58 ± 0.90 ^Cb^	29.75 ± 0.90 ^CDa^	(SD)	<0.0001
14 days	25.49 ± 0.95 ^Bb^	25.31 ± 0.95 ^Cb^	31.57 ± 0.95 ^BCa^	(ST)	<0.0001
21 days	25.69 ± 0.95 ^Bb^	26.69 ± 1.04 ^BCb^	32.80 ± 0.98 ^ABa^	(SD × ST)	<0.0001
28 days	37.46 ± 1.04 ^Aa^	38.76 ± 1.01 ^Aa^	35.03 ± 0.95 ^Ab^		

^1 A–E^ Means followed by different capital letters in the same column differ from each other by the Tukey test (*p* < 0.05). ^2 a–c^ Means followed by different lowercase letters on the same line differ from each other by the Tukey test (*p* < 0.05). ST—storage time/20 samples/group, SD—severity degree/20 samples/group.

**Table 6:** Sensory scores of fresh sausages produced with breast meat from Ross (AP95) broiler chickens without myopathies and affected by wooden breast myopathy, obtained in a sensory test with untrained tasters using a nine-point hedonic scale.

Sensory Attribute	Normal	Moderate	Severe	*p*-Value
Appearance	7.02 ± 0.15	6.94 ± 0.18	6.93 ± 0.18	0.9096
Aroma	7.36 ± 0.14	7.16 ± 0.16	7.07 ± 0.17	0.3857
Taste	7.76 ± 0.11	7.46 ± 0.16	7.51 ± 0.16	0.2043
Texture	7.17 ± 0.15	7.52 ± 0.15	7.32 ± 0.16	0.2611
Overall acceptance	7.43 ± 0.12	7.29 ± 0.15	7.28 ± 0.17	0.6948

## Data Availability

This data can be found here: https://repositorio.unesp.br/bitstream/handle/11449/191219/oliveira_rf_dr_jabo.pdf?sequence=5&isAllowed=y.
